# Acquired resistance to LY2874455 in *FGFR2*-amplified gastric cancer through an emergence of novel *FGFR2-ACSL5* fusion

**DOI:** 10.18632/oncotarget.14788

**Published:** 2017-01-21

**Authors:** Sun Young Kim, Taejin Ahn, Heejin Bang, Jun Soo Ham, Jusun Kim, Seung Tae Kim, Jiryeon Jang, Moonhee Shim, So Young Kang, Se Hoon Park, Byung Hoon Min, Hyuk Lee, Won Ki Kang, Kyoung-Mee Kim, Woongyang Park, Jeeyun Lee

**Affiliations:** ^1^ Division of Hematology-Oncology, Department of Medicine, Samsung Medical Center, Sungkyunkwan University School of Medicine, Seoul, Korea; ^2^ Samsung Genome Institute, Seoul, Korea; ^3^ Department of Pathology and Translational Genomics, Samsung Medical Center, Sungkyunkwan University School of Medicine, Seoul, Korea; ^4^ Division of Gastroenterology, Department of Medicine, Samsung Medical Center, Sungkyunkwan University School of Medicine, Seoul, Korea; ^5^ Department of Molecular Cell Biology, Sungkyunkwan University School of Medicine, Seoul, Korea

**Keywords:** gastric cancer, FGFR2 amplification, targeted therapy

## Abstract

**Background:**

Fibroblast growth factor 2 (*FGFR2*) amplification, occurring in ~2–9% of gastric cancers (GC), is associated with poor overall survival.

**Results:**

RNA sequencing identified a novel *FGFR2-ACSL5* fusion in the resistant tumor that was absent from the matched pre-treatment tumor. The *FGFR2*-amplified PDC line was sensitive to FGFR inhibitors whereas the PDC line with concomitant FGFR2 amplification and FGFR2-ACSL5 fusion exhibited resistance. Additionally, the *FGFR2*-amplified GC PDC line, which was initially sensitive to FGFR2 inhibitors, subsequently also developed resistance.

**Materials and Methods:**

We identified an *FGFR2*-amplified patient with GC, who demonstrated a dramatic and long-term response to LY2874455, a pan-FGFR inhibitor, but eventually developed an acquired LY2874455 resistance. Following resistance development, an endoscopic biopsy was performed for transcriptome sequencing and patient-derived tumor cell line (PDC) establishment to elucidate the underlying molecular alterations.

**Conclusions:**

FGFR inhibitors may function against *FGFR2*-amplified GC, and a novel *FGFR2-ACSL5* fusion identified by transcriptomic characterization may underlie clinically acquired resistance.

**Implications for Practice:**

Poor treatment response represents a substantial concern in patients with gastric cancer carrying multiple *FGFR2* gene copies. Here, we show the utility of a general FGFR inhibitor for initial response prior to treatment resistance and report the first characterization of a potential resistance mechanism involving an *FGFR2-ACSL5* fusion protein.

## INTRODUCTION

Fibroblast growth factor (FGF) receptor family members (FGFR1–4) belong to the receptor-tyrosine kinase superfamily. Binding of the FGF ligand to the receptor induces FGF dimerization to form the FGFR complex, leading to kinase activation and auto-phosphorylation of multiple tyrosine residues in the cytoplasmic domain of the receptor. In turn, this results in downstream signaling activation of the phosphoinositide 3-kinase (PI3K)-AKT and mitogen-activated protein kinase-extracellular signal-regulated kinase pathways [1]. The frequency of *FGFR2* amplification in gastric cancer (GC) varies from 2 to 9% [2–5], with *MET* and *HER2* amplifications being mutually exclusive with *FGFR2* amplification. For example, in a large *FGFR2* amplification screening study, Kilgour and colleagues screened 764 GC samples (408 from Caucasian patients and 356 from Korean patients) and found that *FGFR2* amplification was slightly more common among the Caucasian patients (7.4%, 30/408) than among the Korean patients (4.2%, 15/356) [6]. In particular, *FGFR2* amplification was associated with a diffuse histological subtype among the Korean patients. Furthermore, *FGFR2* amplification was also associated with significantly shorter overall survival in both the Caucasian [Hazards ratio (HR) = 2.37; 95% confidence interval (CI) 1.6–3.5; *P* = 0.0001)] and Korean (HR = 2.33; 95% CI 1.28–4.25; *P* = 0.0129) cohorts [6].

Notably, preclinical results have demonstrated robust anti-tumor efficacies of various FGFR-selective, small-molecule inhibitors such as AZD4547, BGJ398, and LY2874455 in *FGFR2-*amplified GC cell lines [7]. Consequently, several phase II trials with FGFR-selective, small-molecule inhibitors and antibodies are currently being conducted to test the efficacy of FGFR inhibition in this subset of patients with GC [8]. Recently, a randomized phase II trial comparing AZD4547 to paclitaxel as second-line treatment for GC and gastroesophageal-junction cancer harboring *FGFR2* amplification or polysomy reported no additional benefit from AZD4547 in terms of response rate, compared with the AZD4547+paclitaxel arm (SHINE;ClinicalTrials.gov identifier: NCT01457846) [9]. Although the results from this trial suggest that the effects of FGFR2 inhibitors may not complement other treatments in *FGFR2*-amplified GC, they do not rule out the possibility of their potential benefit; thus, the final results from ongoing trials will require careful evaluation. Furthermore, a recent phase I study with FPA144, an FGFR2b isoform-selective monoclonal antibody, in patients with FGFR2b+ GC, demonstrated a response rate of 33% (3 of 9) [10]. Some factors posited as responsible for the development of acquired resistance to FGFR2 inhibition, such as those evinced in these patients, are mTOR [11], EGFR [12, 13], HER [14, 15], MET [14], and loss of PTEN [12], based on cell line data. However, the actual mechanism of acquired resistance to FGFR2 inhibitors in patients has not yet been reported. To understand the mechanism for the acquired resistance, the analysis of pre- and post-treatment biopsy material might allow the detection of different genomic alterations via next-generation sequencing.

In this study, we report the clinical application of genomic profiling in a patient with metastatic GC and peritoneal seeding, who harbored *FGFR2* amplification. The patient originally achieved a durable response to LY2874455 but eventually developed acquired resistance while on treatment. At the time when drug resistance developed, the patient consented to undergo re-biopsy of the progressive tumor at the primary site for transcriptome sequencing. Via RNA sequencing, we identified a newly emerged *FGFR2*-*ACSL5* fusion that was responsible for drug resistance to LY2874455 in *FGFR2*-amplified GC.

## RESULTS

### Case presentation

A 37-year-old female presented with a complaint of weight loss and vomiting. Esophagogastroduodenoscopy (EGD) showed diffuse, reddish mucosal change with erythematous, friable, hypertrophic folds along the entire stomach, indicating advanced Bormann type-4 GC with invasion into the duodenum. An endoscopic biopsy was performed and pathological examination of the stomach showed signet ring cell carcinoma. Laparoscopic surgery was performed with the intent of curative surgery, which revealed disseminated peritoneal seeding. The surgery was precluded with opening and closure. The tumor was HER2-negative.

**Figure 1:**
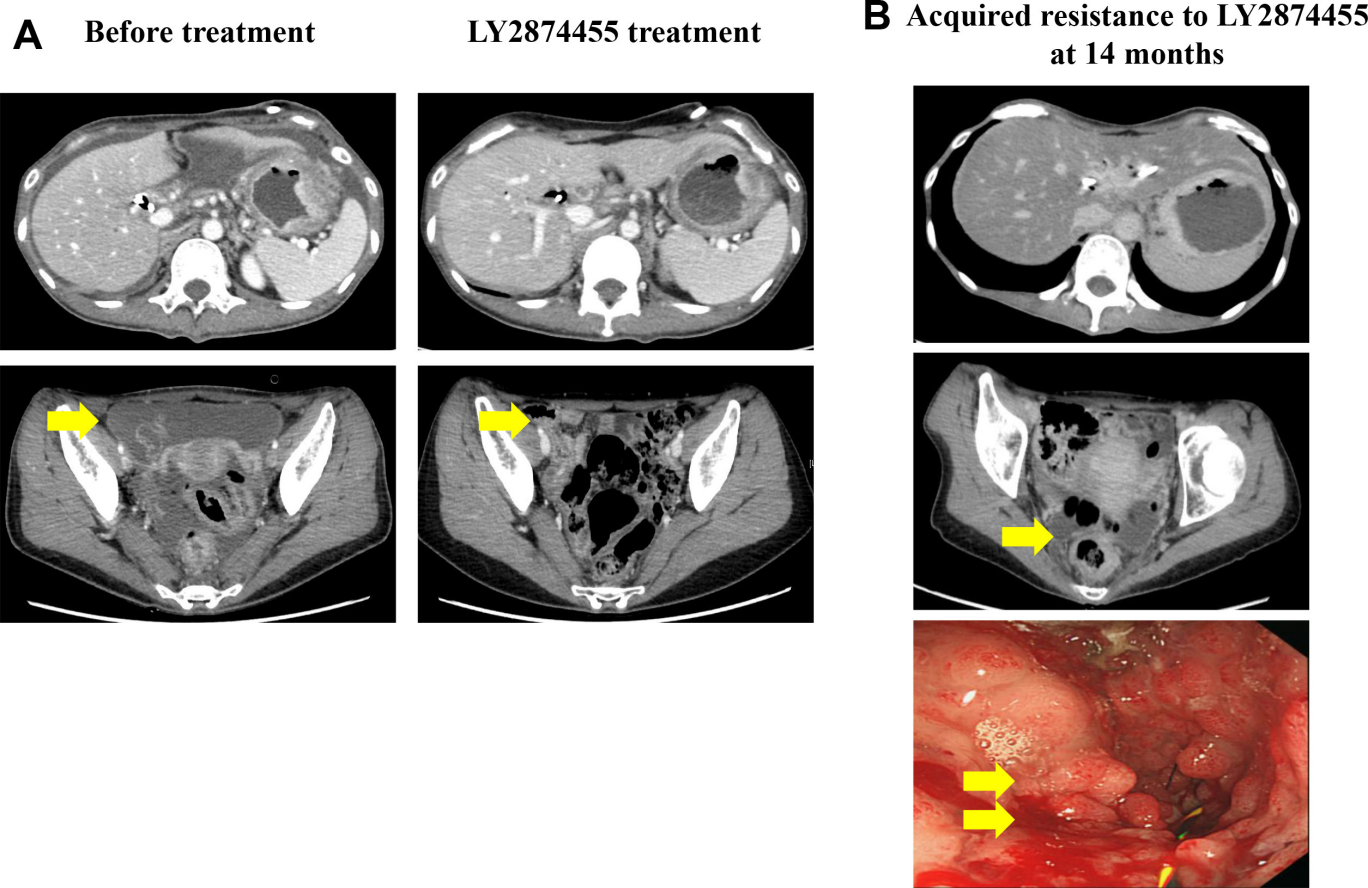
(**A**) Computed tomography scans prior to and following LY2874455 treatment. The patient exhibited peritoneal seeding and primary gastric cancer mass before treatment. (**B**) Upon the development of resistance to LY2874455, the patient developed peritoneal seeding and an obstructive gastric mass.

The patient underwent eight cycles of oxaliplatin and capecitabine therapy and achieved a partial response. At progression, the patient received 12 cycles of 5-fluorouracil and irinotecan (FOLFIRI) every 2 weeks, achieving partial response as the best response. During second-line chemotherapy, a biliary stent was inserted because the biliary tract was obstructed owing to cancerous invasion of the ampulla of Vater. Immediately after the last cycle of FOLFIRI, tumorous obstruction developed because of cancer progression and a stent was inserted to alleviate obstructive symptom. As third-line palliative chemotherapy, eight cycles of weekly docetaxel, with infusion on days 1 and 8, were administered every 3 weeks. The patient was enrolled in a phase I trial of LY2874455, a pan-FGFR inhibitor, that inhibits autophosphorylation of FGFR1–4. Her symptoms, including oral intake, improved significantly shortly after being treated with LY287445, with complete disappearance of ascites. Her response criteria, however, were categorized as reflecting stable disease per the RECIST 1.1 guidelines (Figure 1A).

After 14 months on LY2874455, the patient complained of an abrupt onset of upper gastrointestinal obstructive symptoms, which prompted us to perform a follow-up EGD that showed tumor ingrowth into the stent, causing near-total obstruction (Figure 1B). At this time, the patient consented to transcriptomic profiling for full genomic testing. To facilitate this, upper gastrointestinal stent re-insertion and endoscopic biopsy were performed followed by total RNA sequencing and targeted amplification at the time of acquired resistance to LY2874455.

### Association of an *FGFR2-ACSL5* Fusion with Acquired Resistance to an FGFR Inhibitor

**Figure 2:**
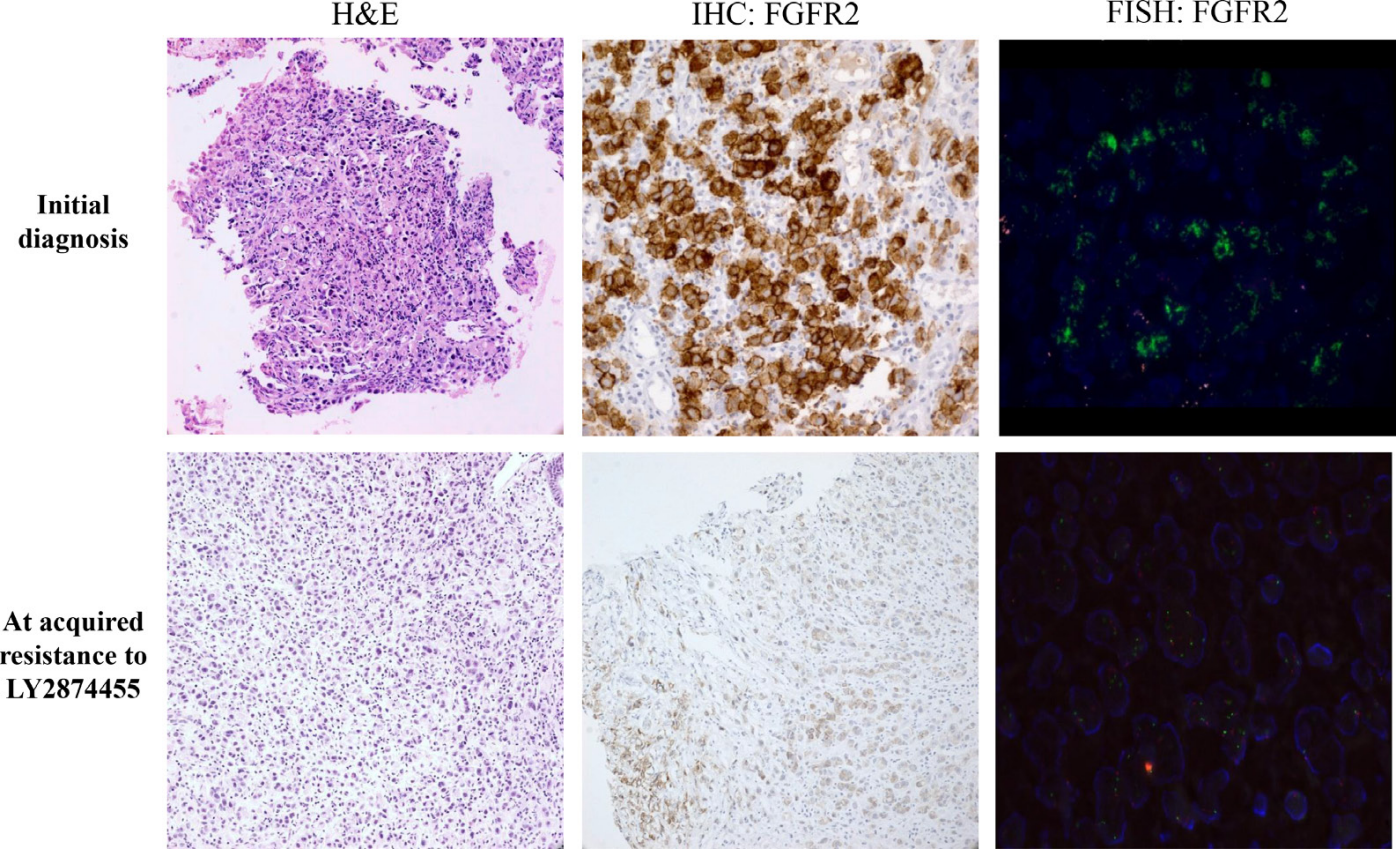
Pathology (hematoxylin & eosin staining, H&E), IHC, and FISH results in primary tumor tissues at the time of diagnosis (upper row) and at the time of acquired resistance to LY2874455 (lower row)

**Figure 3:**
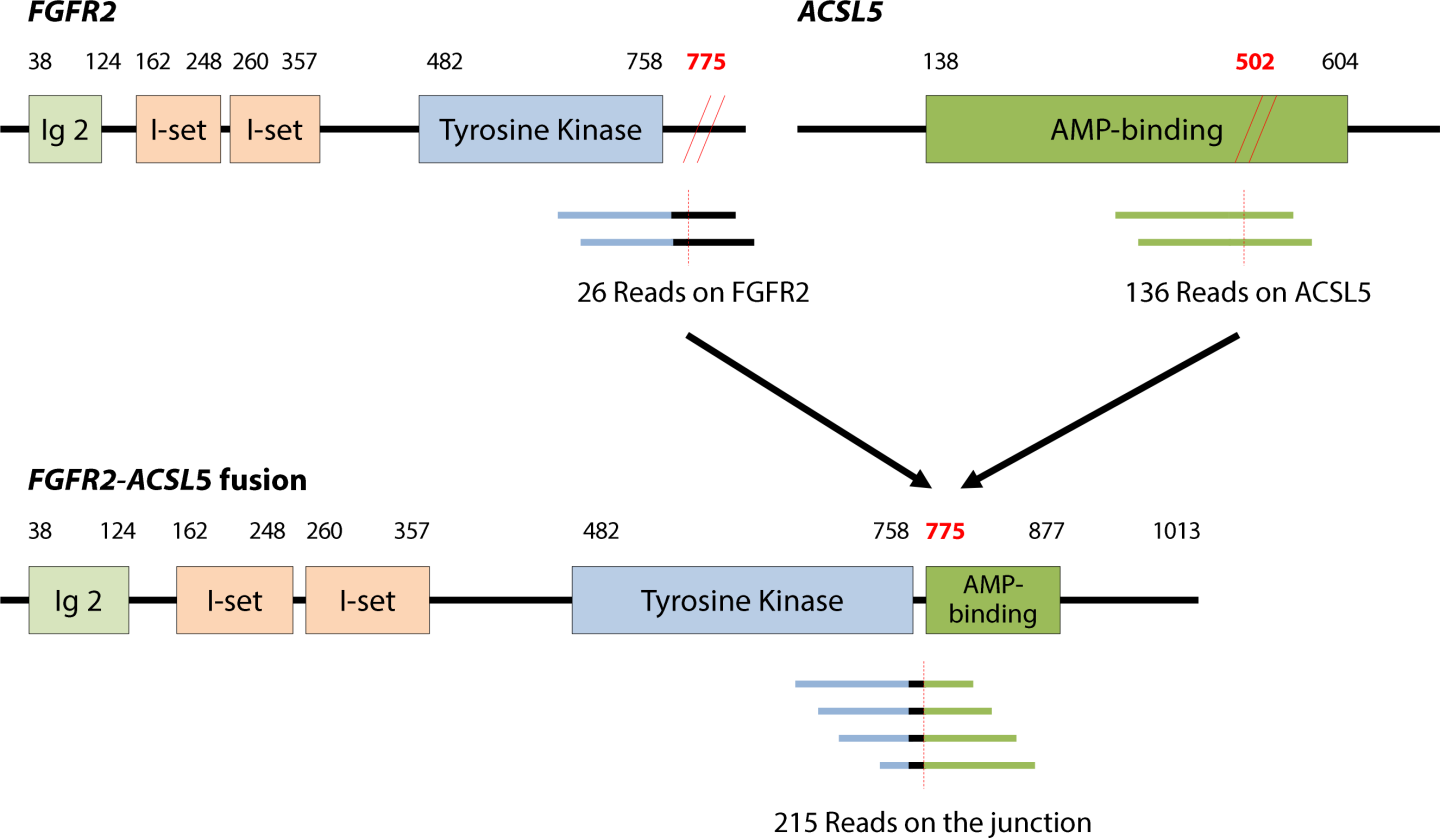
*FGFR2-ACSL5* fusion transcript identified by RNA sequencing The numbers of supporting reads of wild-type and fusion transcripts obtained are indicated below the respective diagrams. The number of supporting reads for the fusion junction suggested that the fusion form was dominantly expressed in the post-progression cancer. Expression of the *FGFR2-ACSL5* fusion transcript was confirmed by qRT-PCR in the post-treatment tumor tissue, but no fusion transcripts were detected in the initial tumor tissue.

Both baseline tumor and tumor tissue at acquired resistance exhibited *FGFR2* amplification, as determined by fluorescence *in situ* hybridization (FISH) and immunohistochemistry (IHC) staining for FGFR2, which showed strong positivity in both the membrane and cytoplasm of tumor cells (Figure 2). Both tumor specimens were from primary GC tissue. Although *FGFR2* amplification was present in both pre- and post-resistance specimens, the average gene copy number ascertained by FISH was 52.5 copies at pre-treatment biopsy (Figure 2), whereas 2.5 copies were detected at resistance. Accordingly, the FGFR2 protein overexpression detected by IHC was present in both specimens, although the intensity decreased in the post-treatment biopsy. Targeted sequencing of pre and post biopsy specimens demonstrated no FGFR2 mutations or aberrations other than FGFR2 amplification (*data not shown*). Via RNA sequencing, we identified a novel *FGFR2*-*ACSL5* fusion in the tumor upon acquired resistance (Figure 3). The fusion mRNA product started from the beginning of *FGFR2* (NM_022970) to the 774^th^ amino acid, with the 775^th^ amino acid of the fusion product corresponding to the 502^nd^ codon of *ACSL5* (NM_016234). The fusion product contained Ig2, I-set, a tyrosine kinase domain from FGFR2, and a truncated AMP-binding domain from ACSL5. Furthermore, an in-house-developed, fusion-read validation procedure showed that 215 supporting reads exactly matched the fusion junction, whereas 26 and 136 reads supported expression of the wild-type *FGFR2* and *ACSL5* genes, respectively. The larger number of reads supporting the gene fusion indicated that the fused form of FGFR2 exhibited elevated expression in the resistant tumor. By quantitative reverse transcription-polymerase chain reaction (qRT-PCR) analysis, we confirmed the presence of markedly elevated levels of *FGFR2*-*ACSL5* fusion transcripts in the post-treatment tumor, whereas no such expression was identified in the pre-treatment, baseline tumor specimen (Figure 3).

The *FGFR2* gene expression from the patient was significantly higher when compared with that from the GC cohort reported in a publically available database (outlier statistic: 3.156, Supplementary Figure 1). In our study, *FGFR2* was the most up-regulated gene among the receptor tyrosine kinases considered, indicating that overexpression of the *FGFR2* fusion transcript played an important role in the patient's acquired resistance (Supplementary Figure 2). Out of the 20 most up-regulated pathways in the patient, three pathways were relevant to the PI3K-AKT-mTOR axis (the PID ARF6 pathway, the BioCarta AKT pathway, and the PID PI3KCI pathway; Supplementary Figure 3). Notably, phosphorylated FGFR2 can activate the PI3K and AKT pathways through the adapter protein FSR2 [16]. According to the ACRG molecular classifications of GC [17], this patient's tumor was of the mesenchymal subtype (Supplementary Figure 4). However, it was unclear whether the up-regulation of these pathways in the tumor was directly resulted from high expression of the *FGFR2*-*ASCL5* fusion product.

**Figure 4:**
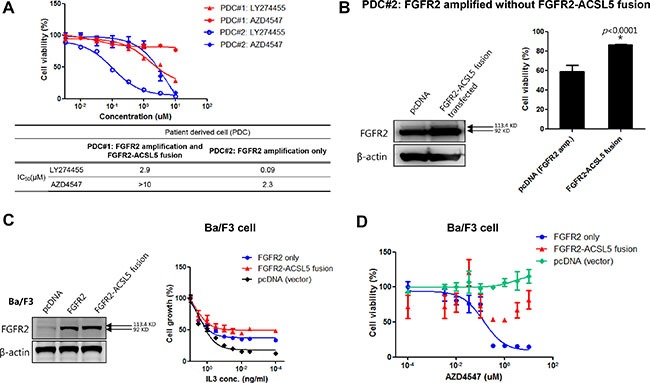
**(A)** Cell viability assay of LY274455 and AZD4547 treatment in PDC#1 cells, which were derived from FGFR2-amplified GC with the *FGFR2-ACSL5* fusion; (**B**) Immunoblot analysis of FGFR2-ACSL5 fusion proteins expressed in Ba/F3 cells (left panel); Growth of *FGFR2-ACSL5*-expressing cells with various concentrations of IL3 (right panel). Results are shown as percentage of growth at the indicated concentration compared to growth in 1 ng/mL IL3. (**C**) Cell viability assay of AZD4547 treatment when the *FGFR2*-*ACSL5* fusion protein was introduced into Ba/F3 cells; (**D**) PDC#2 cells, generated from another FGFR2-amplified GC, were transfected with pcDNA only or the *FGFR2*-*ACSL5* fusion construct, and cell viability was measured after treatment with 1 μM AZD4547. FGFR2 and FGFR2-ACSL5 fusion protein expression was detected by immunoblotting.

Therefore, to further examine the function of the fusion protein, we established a patient-derived tumor cell line that carried 2.5 copies of the FGFR2 amplification and the concomitant FGFR2-ACSL5 fusion (PDC#1) and established PDC#2 from a different FGFR2-amplified without fusion patient with GC who was chemotherapy naïve. This patient had not received any FGFR-targeted treatment at the time of PDC establishment. (Supplementary Figure 5, PDC#2). As anticipated, PDC#1 was resistant to both LY274455 and AZD4547 (2.9 μM and > 10 μM of IC_50_ value, respectively) compare to PDC#2 which is FGFR2 amplified only (Figure 4A). We then transfected PDC#2 with the *FGFR2-ACSL5* fusion construct and estimated cell viability after treatment of 1 μM AZD4547. The introduction of the *FGFR2-ACSL5* fusion to the *FGFR2*-amplified PDC line that was sensitive to FGFR inhibitors resulted in a relative resistance to FGFR inhibitors (cell viability of 86.6% *vs* 59.2%; 1 μM AZD4547, *P* < 0.0001; Figure 4B). To examine the effects of the *FGFR2-ACSL5* fusion in isolation from FGFR2 amplification, we transfected Ba/F3 cells with a *FGFR2-ACSL5* fusion construct (Figure 4C). This murine hematopoietic cell line is IL3-dependent and was transformed to IL3 independence by the expression of the *FGFR2-ACSL5* fusion protein (Figure 4C, right panel). Furthermore, isogenic Ba/F3-*FGFR2-ACSL5* cells were resistant to AZD4547 whereas Ba/F3-*FGFR2* cells exhibited AZD4547 sensitivity (Figure 4D). Taken together, presence of the *FGFR2-ACSL5* fusion was associated with resistance to AZD4547, the FGFR2 inhibitor currently used as treatment in patients with *FGFR2*-amplified tumors.

## DISCUSSION

First, this case clearly shows that patients with GC and *FGFR2* amplification may durably respond to FGFR-inhibitor therapy, despite the lack of observed additional benefits provided by AZD4547 compared to those by AZD4547 (+) paclitaxel in a recent randomized phase II trial [9]. However, the results from this trial do not preclude the possibility of potential benefits from FGFR2 inhibitors in *FGFR2*-amplified GC, especially in light of our index case. Notably, the patient failed on three prior regimens before enrollment into the LY2874455 (pan-FGFR inhibitor) trial, wherein the patient, whose tumor was characterized as exhibiting FGFR2 protein overexpression by IHC as well as high-copy-number *FGFR2* amplification, responded for over 1 year.

Recently, *FGFR1-TACC1* and *FGFR3-TACC3* fusions have been identified in approximately 3% of glioblastoma multiforme tumor samples [16] and *FGFR3-TACC3* fusions were identified in a subset of bladder carcinomas [17]. Preclinical findings suggest that patients with glioblastoma multiforme and *FGFR*–*TACC* gene fusions may benefit from targeted FGFR kinase inhibition [18, 19]. In addition, four *FGFR* fusion-positive patients were identified in the MI-ONCOSEQ study, with *FGFR2*-*BICC1* rearrangements occurring in two cases of cholangiocarcinoma, a *FGFR2*-*AFF3* fusion occurring in breast cancer, and a *SLC45A3*-*FGFR2* fusion occurring in prostate cancer. None of these four patients were treated with FGFR-directed therapy, which emphasizes the importance of sequencing to identify non-targeted, potentially clinically relevant rearrangements, as well as the availability of clinical trials after obtaining sequencing results.

In the current study, a novel *FGFR2*-*ASCL5* fusion was not detected at the baseline but was acquired following the development of resistance to an FGFR-inhibitor. *FGFR2* amplification at resistance was detectable with concomitant formation of the *FGFR2*-*ASCL5* gene fusion. Consistent with this, introduction of the fusion gene conferred resistance to various FGFR2 inhibitors in cell culture. However, although the specific function of this novel gene fusion is currently unknown, the fusion product contains Ig2, I-set, the FGFR2 tyrosine kinase domain, and a truncated AMP-binding domain from ASCL5. We found via RNA sequencing that the PIK3-AKT-mTOR pathway was highly activated in fusion-expressing cells; further experiments are needed to determine whether the FGFR2-ASCL5 fusion protein directly activates this pathway (Supplementary Figure 3). Rare recurrent FGFR2 fusions have been reported in GC, although FGFR2-ASCL5 fusion has not been published yet [20].

Notably, the patient tumor was classified as a mesenchymal subtype according to our recent report on molecular classifications in GC [17]. In comparison, in a comprehensive molecular classification study involving 300 patients with GC, *FGFR2* amplification was identified in 1.2% of MSS/TP53-intact subtype tumors, 3.0% of MSS/TP53-mutant subtype tumors, and 4.9% of mesenchymal-subtype tumors [3].

Thus, based on our findings, *FGFR2* amplification represents a promising target especially within the mesenchymal subtype of GC, which is predominantly diffuse and found in females and younger patients. The impact of *FGFR2* amplification on treatment outcomes in each of the four GC subtypes needs to be defined. Here, we found that one of the resistance mechanisms against treatment efficacy utilized by *FGFR2*-amplified GC comprises *FGFR2-ASCL5* fusion. This discovery and the RNA sequencing strategy applied might in turn provide clues toward developing specific modalities for subsequent, post-resistance treatment of GC.

## MATERIALS AND METHODS

### Ethics statement

The patient was enrolled in the molecular screening program, a prospective molecular-profiling study conducted at SMC (trial registration ID: NCT02141152), as described [18, 19]. The study participant provided written informed consent before study entry. Briefly, patients with metastatic solid tumors who might have been eligible for clinical trial enrollment were eligible to enter the study. The phase I trial of LY2874455 will be reported elsewhere.

### Analysis of RNA sequencing, gene expression, and pathway regulation

Fastq files from RNA sequencing of the patient samples were mapped to human genome reference (hg19) according to the bowtie method [21]. Tophat was used to generate read counts per gene [22] and FusionMap was used to discover fusion candidates. An in-house method was developed for inferring the actual read counts supporting the fusion junction and the wild-type sequences of the respective genes. Briefly, we normalized the patient gene expression data with publically available GC data. Gene expression data from the STAD TCGA project (tumor = 238, normal = 33) were used for normalization purposes. The statistical algorithm COMBAT was applied to reduce the impact of platform and batch effects on data analysis [23]. After reducing the batch effect, we standardized the patient gene expression levels using the mean and standard deviations of normal gastric -tissue gene-expression values. Individualized pathway alteration scores were obtained following the IPAS method [24].

### FGFR2 fusion confirmation by real-time qRT-PCR

qRT-PCR assays were conducted to validate expression of the fusion transcripts. Briefly, total RNA was extracted from fresh tumor tissue (tumor content > 70%) of resistant tumor biopsies and from four formalin-fixed paraffin-embedded tissue sections (4-μm thick) of primary tumor biopsies, using the High Pure RNA Paraffin Kit (Roche Diagnostics, Mannheim, Germany). After RNA extraction, we performed an additional DNase-treatment step. RNA concentrations were measured using a NanoDrop 8000 (Thermo-Scientific, Wilmington, DE) and RNAs were reverse transcribed using a Superscript III First-Strand Synthesis System (Invitrogen, Carlsbad, CA).

qRT-PCR was conducted using fusion-specific primers (5′-CCA GGT AAT GTA AAT GTA CAG CCA CCT GTG CAT TCT GTT TGA CCA TAA GCT TCA TAC ACC TCA TTG GTT GTG AGA GTG AGA ATT CGA TCC AAG TCT TCT ACC AAC TGC-3′; bold, primers; red, probe) with the TaqMan Gene Expression Master Mix (Part No. 4369016, Applied Biosystems, Foster, City, CA) and a Custom TaqMan Gene Expression *FGFR2-ACSL5* fusion assay (Assay ID AI39SUU; Applied Biosystems). GAPDH (Assay ID Hs99999905_m1; Applied Biosystems) was used as an internal control.

### FISH and IHC against FGFR2

FISH was performed using a ZytoLight SPEC FGFR2/CEN 10 Dual Color Probe (Z-2122–200, Zytovision, Bremerhaven, Germany). Respective DNA probe sets were applied to 1 μm-thick tumor sections and incubated overnight at 37°C. We counted the hybridization signals in 20 nuclei per sample under a fluorescence microscope. All overlapping nuclei were excluded and only nuclei with a distinct nuclear border were evaluated. The *FGFR2* gene was considered amplified when the *FGFR2*:*CEP17* FISH-signal ratio was ≥ 2.0.

IHC was performed for FGFR2 on formalin-fixed, paraffin-embedded, 4-μm-thick tissue sections with a primary mouse monoclonal anti-human FGFR2 antibody (MAB6841, R&D Systems, Minneapolis, MN). The tissue sections were deparaffinized and rehydrated. After antigen retrieval and endogenous peroxidase blocking, the samples were incubated with primary antibody for 15 min. A BOND-MAX autoimmunostainer (Leica Biosystems, Melbourne, Australia) with Bond™ Polymer Refine Detection, DS9800 (Vision Biosystems, Melbourne, Australia), was used according to the manufacturer's protocol.

### Patient-derived tumor cell culture

Malignant ascites were collected from patients, as previously described [25]. Collected effusions (1–5 L) were divided into 50-mL tubes, centrifuged at 1500 rpm for 10 min, and washed twice with phosphate-buffered saline. Cell pellets were resuspended in culture medium and plated into 75-cm^2^ culture flasks. Extracted cells were cultured in Roswell Park Memorial Institute (RPMI) medium supplemented with 10% fetal bovine serum, 0.5 g/mL hydrocortisone (Sigma Aldrich, St. Louis, MO), 5 g/mL insulin (PeproTech, Rocky Hill, NJ), and 5 ng EGF (PeproTech). After pathological confirmation, cells were seeded at 1 × 10^6^ cells/10 mm dishes or 5000 cells/well in 96-well plates. Treated cells were incubated for 72 hours at 37°C in 5% carbon dioxide. These conditions were used when analyzing immunoblotting and cell proliferation-inhibition results, which were run in triplicate. Inhibition of tumor-derived cell line proliferation was determined using the CellTiter-Glo^®^ Reagent (Promega, Madison, WI).

### Generation of FGFR2-ACSL5 constructs

Full-length *FGFR2-ACSL5* cDNAs were constructed by splicing partial fragments (Supplementary Figure 6) generated from a MegaMan cDNA library (Stratagene, La Jolla, CA). The complete *FGFR2-ACSL5* cDNAs were inserted into pcDNA3.1 (Invitrogen), using the *Eco*RI/*Pme*I sites. Sequence analysis was performed using an ABI 3730xl DNA analyzer (Thermo Fisher Scientific, Rockford, IL) to verify the open reading frames of the constructs.

### Cell lines and transfections

Ba/F3 cells were obtained from the RIKEN BRC CELL BANK (Ibaraki, Japan) and cultured in RPMI 1640 supplemented with 10% fetal bovine serum and 1 ng/mL recombinant IL3 (R&D Systems). Then, 5 × 10^6^ cells were electroporated with 2.5 mg pcDNA3.1/*FGFR2*-constructs, pcDNA3.1/*FGFR2-ACSL5* fusion constructs, or parental pcDNA3.1, using the Nucleofector system (Lonza, Basel, Switzerland). The cells were selected in medium containing G418 for 2 weeks and the expression of the full-length fusion transcripts was confirmed by immunoblotting. For detection of cell viability, 5 × 10^3^ Ba/F3 cells per well were plated in quadruplicate in 96-well plates with various concentrations of IL3 or AZD4547. After 72 hours, cell viability was determined using CellTiter-Glo.

### Immunoblot analysis

Total proteins from cells were isolated using RIPA buffer (Sigma-Aldrich) containing a protease inhibitor cocktail (Roche) and phosphatase inhibitor cocktail (Roche), and protein concentrations were determined according to the Bradford procedure, using a Quick Start Bradford Protein Assay (Bio-Rad, Hercules, CA). For immunoblotting, 30 μg protein was subjected to 10% sodium dodecyl sulfate-polyacrylamide gel electrophoresis and electro-transferred onto nitocellulose membranes. The membranes were blocked with 5% nonfat dry milk in Tris-buffered saline containing 0.1% v/v Tween 20 and probed overnight at 4°C with specific antibodies: anti-FGFR2 from Abcam (Cambridge, UK) and anti-beta actin from Sigma Aldrich. Horseradish peroxidase-conjugated anti-rabbit or mouse IgG (Vector, Burlingame, CA) was used as a secondary antibody; signals were detected by chemiluminescence using the ECL Western Blotting Substrate (Thermo Scientific) and visualized using ImageQuant LAS-4000 (Fujifilm, Tokyo, Japan).

## SUPPLEMENTARY MATERIALS FIGURES



## References

[1] Katoh M, Katoh M (2006). FGF signaling network in the gastrointestinal tract (review). Int J Oncol.

[2] Xie L, Su X, Zhang L, Yin X, Tang L, Zhang X, Xu Y, Gao Z, Liu K, Zhou M, Gao B, Shen D, Zhang L (2013). FGFR2 gene amplification in gastric cancer predicts sensitivity to the selective FGFR inhibitor AZD4547. Clin Cancer Res.

[3] Lee J, Lim DH, Kim S, Park SH, Park JO, Park YS, Lim HY, Choi MG, Sohn TS, Noh JH, Bae JM, Ahn YC, Sohn I (2012). Phase III trial comparing capecitabine plus cisplatin versus capecitabine plus cisplatin with concurrent capecitabine radiotherapy in completely resected gastric cancer with D2 lymph node dissection: the ARTIST trial. J Clin Oncol.

[4] Matsumoto K, Arao T, Hamaguchi T, Shimada Y, Kato K, Oda I, Taniguchi H, Koizumi F, Yanagihara K, Sasaki H, Nishio K, Yamada Y (2012). FGFR2 gene amplification and clinicopathological features in gastric cancer. Br J Cancer.

[5] Deng N, Goh LK, Wang H, Das K, Tao J, Tan IB, Zhang S, Lee M, Wu J, Lim KH, Lei Z, Goh G, Lim QY (2012). A comprehensive survey of genomic alterations in gastric cancer reveals systematic patterns of molecular exclusivity and co-occurrence among distinct therapeutic targets. Gut.

[6] Fuchs CS, Tomasek J, Yong CJ, Dumitru F, Passalacqua R, Goswami C, Safran H, dos Santos LV, Aprile G, Ferry DR, Melichar B, Tehfe M, Topuzov E (2014). Ramucirumab monotherapy for previously treated advanced gastric or gastro-oesophageal junction adenocarcinoma (REGARD): an international, randomised, multicentre, placebo-controlled, phase 3 trial. Lancet.

[7] Kunii K, Davis L, Gorenstein J, Hatch H, Yashiro M, Di Bacco A, Elbi C, Lutterbach B (2008). FGFR2-amplified gastric cancer cell lines require FGFR2 and Erbb3 signaling for growth and survival. Cancer Res.

[8] Lee J, Ou SH (2013). Towards the goal of personalized medicine in gastric cancer--time to move beyond HER2 inhibition. Part II: Targeting gene mutations and gene amplifications and the angiogenesis pathway. Discov Med.

[9] Lee J, Sohn I, Do IG, Kim KM, Park SH, Park JO, Park YS, Lim HY, Sohn TS, Bae JM, Choi MG, H Lim do, Min BH (2014). Nanostring-based multigene assay to predict recurrence for gastric cancer patients after surgery. PLoS One.

[10] Lee J BJC, Rha S.Y, Bang YJ (2016). Antitumor activity and safety of FPA144, an ADCC-enhanced, FGFR2b isoform-selective monoclonal antibody, in patients with FGFR2b+ gastric cancer and advanced solid tumors. J Clin Oncol.

[11] Gozgit JM, Squillace RM, Wongchenko MJ, Miller D, Wardwell S, Mohemmad Q, Narasimhan NI, Wang F, Clackson T, Rivera VM (2013). Combined targeting of FGFR2 and mTOR by ponatinib and ridaforolimus results in synergistic antitumor activity in FGFR2 mutant endometrial cancer models. Cancer Chemother Pharmacol.

[12] Herrera-Abreu M, Garcia-Murilla I, Pearson A, Shnyder S, Knowles M, Turner NC (2012). 163 functional screens to identify mechanisms of resistance to FGFR inhibitors in FGFR amplified and mutated cell lines. Eur J Cancer.

[13] Herrera-Abreu MT, Pearson A, Campbell J, Shnyder SD, Knowles MA, Ashworth A, Turner NC (2013). Parallel RNA interference screens identify EGFR activation as an escape mechanism in FGFR3-mutant cancer. Cancer Discov.

[14] Harbinski F, Craig VJ, Sanghavi S, Jeffery D, Liu L, Sheppard KA, Wagner S, Stamm C, Buness A, Chatenay-Rivauday C, Yao Y, He F, Lu CX (2012). Rescue screens with secreted proteins reveal compensatory potential of receptor tyrosine kinases in driving cancer growth. Cancer Discov.

[15] Issa A, Gill JW, Heideman MR, Sahin O, Wiemann S, Dey JH, Hynes NE (2013). Combinatorial targeting of FGF and ErbB receptors blocks growth and metastatic spread of breast cancer models. Breast Cancer Res.

[16] Ahmad I, Iwata T, Leung HY (2012). Mechanisms of FGFR-mediated carcinogenesis. Biochim Biophys Acta.

[17] Cristescu R, Lee J, Nebozhyn M, Kim KM, Ting JC, J WongSS Liu, Yue YG, Wang J, Yu K, Ye XS, Do IG, Liu S (2015). Molecular analysis of gastric cancer identifies subtypes associated with distinct clinical outcomes. Nat Med.

[18] Yi JH, Jang J, Cho J, Do IG, Hong M, Kim ST, Kim KM, Lee S, Park SH, Park JO, Park YS, Kang WK, Lim HY (2015). MerTK is a novel therapeutic target in gastric cancer. Oncotarget.

[19] Lee J (2015). Gastrointestinal malignancies harbor actionable MET exon 14 deletions. Oncotarget.

[20] Stransky N, Cerami E, Schalm S, Kim JL, Lengauer C (2014). The landscape of kinase fusions in cancer. Nat Commun.

[21] Langmead B, Trapnell C, Pop M, Salzberg SL (2009). Ultrafast and memory-efficient alignment of short DNA sequences to the human genome. Genome Biol.

[22] Pollier J, Rombauts S, Goossens A (2013). Analysis of RNA-Seq data with TopHat and Cufflinks for genome-wide expression analysis of jasmonate-treated plants and plant cultures. Methods Mol Biol.

[23] Johnson WE, Li C, Rabinovic A (2007). Adjusting batch effects in microarray expression data using empirical Bayes methods. Biostatistics.

[24] Ahn T, Lee E, Huh N, Park T (2014). Personalized identification of altered pathways in cancer using accumulated normal tissue data. Bioinformatics.

[25] Lee JY, Kim SY, Park C, Kim NK, Jang J, Park K, Yi JH, Hong M, Ahn T, Rath O, Schueler J, Kim ST, Do IG (2015). Patient-derived cell models as preclinical tools for genome-directed targeted therapy. Oncotarget.

