# Supplementary material for: Acquired resistance to LY2874455 in FGFR2-amplified gastric cancer through an emergence of novel FGFR2-ACSL5 fusion
Source: Oncotarget. 2017 Jan 21;8(9):15014–22. doi: 10.18632/oncotarget.14788 (PMC5362463; doi:10.18632/oncotarget.14788)
Supplement: Supplementary file 1 [file oncotarget-08-15014-s001.pdf]

Acquired resistance to LY2874455 in *FGFR2*-amplified gastric cancer through an emergence of novel *FGFR2-ACSL5* fusion

Supplementary Materials

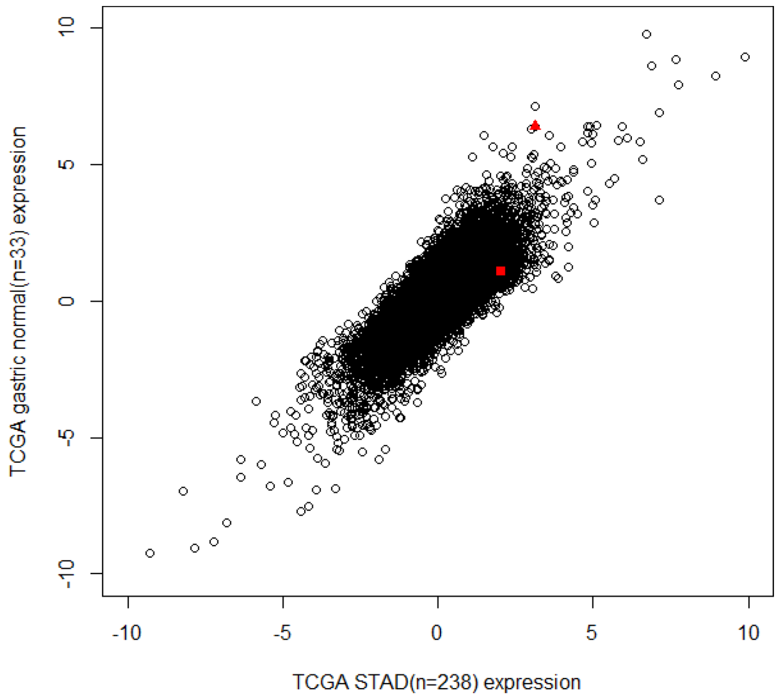

Supplementary Figure 1: Gene expression levels of the patient, compared to publically available expression data. The patient showed high FGFR2 expression compared to that of GC (outlier statistic: 3.16) and normal gastric tissue (outlier statistic: 6.48). The patient showed higher ASCL5 expression than observed in other STAD cancers (outlier statistic: 2.02), but the expression was not significantly higher than normal gastric tissues (outlier statistic: 1.07). FGFR2 and ASCL5 are highlighted with a red triangle and square, respectively.

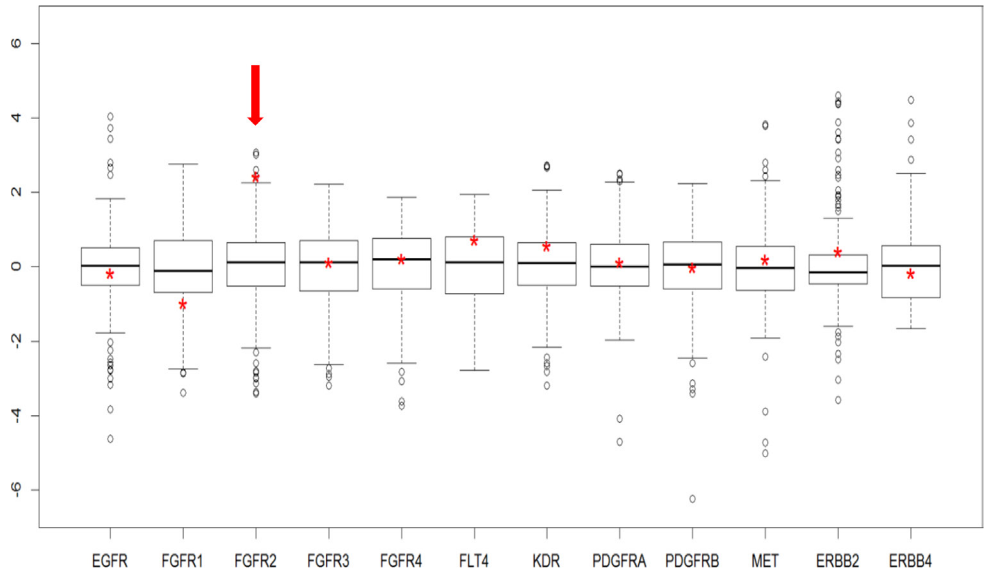

Supplementary Figure 2: The patient's gene expression level is highlighted with a red star. FGFR2 expression was the most up-regulated gene relative to expression levels observed in other GC tissues acquired from TCGA STAD.

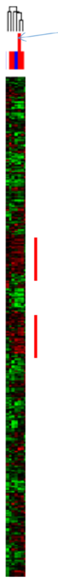

Pathway	Pathway score
REACTOME_REGULATION_OF_BETA_CELL_DEVELOPMENT	1.659
REACTOME_GASTRIN_CREB_SIGNALLING_PATHWAY_VIA_PKC_AND_MAPK	1.479
KEGG_ALDOSTERONE_REGULATED_SODIUM_REABSORPTION	1.338
PID_ENDOTHELINPATHWAY	1.320
KEGG_CHEMOKINE_SIGNALING_PATHWAY	1.100
BIOCARTA_BAD_PATHWAY	1.087
REACTOME_CA_DEPENDENT_EVENTS	1.073
PID_ARF6_PATHWAY	1.054
KEGG_CYTOKINE_CYTOKINE_RECEPTOR_INTERACTION	1.018
BIOCARTA_AKT_PATHWAY	1.015
BIOCARTA_ACH_PATHWAY	1.010
PID_IFNGPATHWAY	1.008
PA165980050-Vemurafenib_Pathway_Pharmacodynamics	1.008
REACTOME_PLATELET_AGGREGATION_PLUG_FORMATION	0.977
PID_IL4_2PATHWAY	0.937
PID_IL6_7PATHWAY	0.929
PID_PI3KCIPATHWAY	0.923
REACTOME_IL_2_SIGNALING	0.895
BIOCARTA_KERATINOCYTE_PATHWAY	0.893
BIOCARTA_CHEMICAL_PATHWAY	0.887

Supplementary Figure 3: Out of 20 most up-regulated pathways in the patient, the AKT and PI3KCI pathways were relatively highly up-regulated.

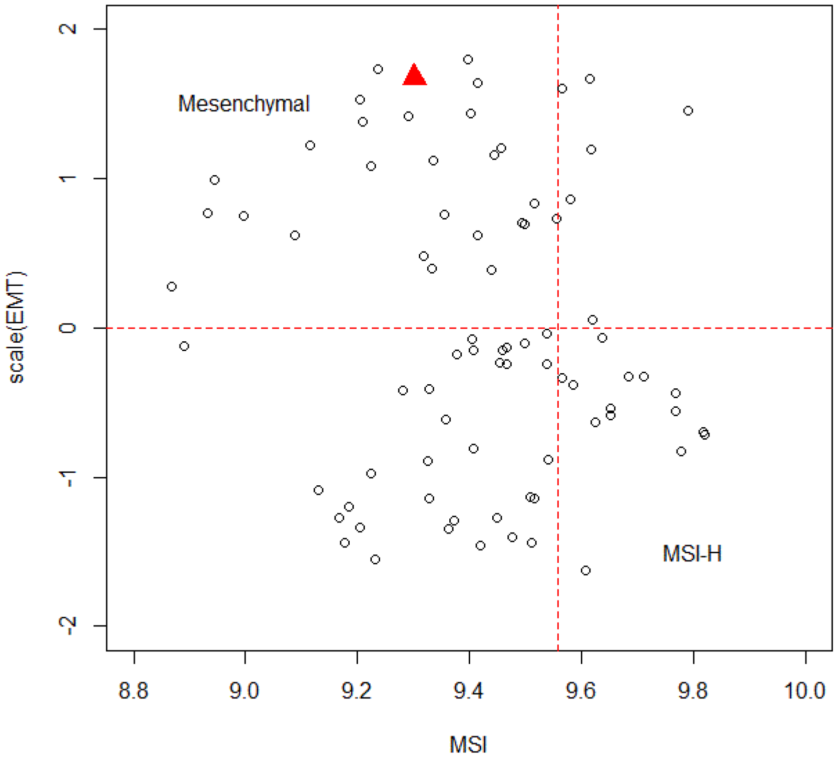

Supplementary Figure 4: Gene signature analysis of epithelial-to-mesenchymal transition (EMT) and microsatellite instability (MSI) distribution is exhibited the patient's molecular subtype is mesenchymal subtype (red triangle).

A PDC#2

Gene Name	Amp.Del	Sig.Ratio	Copy Number	Num.exon	Sig.exon	Location
FGFR2	Amp.	2.06	8.32	23	23	10q26

B

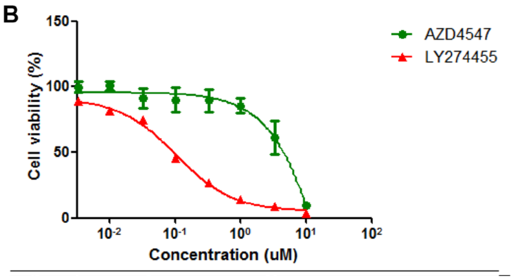

Supplementary Figure 5: Genomic and physiological features of PDC#2. (**A**) Copy number variation result of FGFR2 from the patient tumor. FGFR2 amplification was detected via CNV of targeted sequencing from the patient DNA. (**B**) PDC#2 from FGFR2 amplified patient tumor was sensitive to LY274455 and AZD4547.

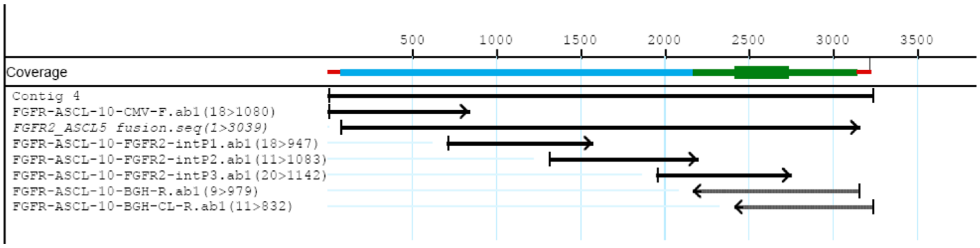

Supplementary Figure 6: Strategy of the *FGFR2-ACSL5* constructs; sequencing alignment graphic of FGFR2 (NM_022970) and ACSL5 (NM_016234) fusion construct inserted in pcDNA 3.1. The putative molecular weight is 113.4 KD.
